# Solventless synthesis of cerium oxide nanoparticles and their application in UV protective clear coatings†

**DOI:** 10.1039/d0ra01710h

**Published:** 2020-04-14

**Authors:** Rubén Álvarez-Asencio, Robert W. Corkery, Anwar Ahniyaz

**Affiliations:** RISE Research Institutes of Sweden, Division of Bioscience and Materials SE-114 86 Stockholm Sweden anwar.ahniyaz@ri.se; KTH Royal Institute of Technology, School of Engineering Sciences in Chemistry, Biotechnology and Health Sweden

## Abstract

Colloidal dispersions of cerium oxide nanoparticles are of importance for numerous applications including as catalysts, chemical mechanical polishing agents and additives for UV protective and anticorrosion coatings. Here, concentrated oleate-coated cerium oxide nanoparticles (CeO_2_ NPs) with a uniform size have been produced by solventless thermolysis of cerium-oleate powder under low pressure at 320 °C and subsequently dispersed in hexane. Unlike any previously reported colloidal synthesis process for ceria nanoparticles, this process does not involve any toxic high boiling point organic solvent that requires subsequent removal at high cost. Although the process is very simple, highly concentrated cerium oxide nanoparticles with more than 17 wt% solid content and 70% of the theoretical yield can be easily obtained. Moreover, the size, shape and crystallinity of cerium oxide nanoparticles can be tailored by changing the thermal decomposition temperature and reaction time. Moreover, the new synthesis route developed in this study allows the synthesis of clean and dispersible ceria nanoparticles at a relatively low cost in a single step. The prepared ceria nanoparticles have an excellent UV absorption property and remain transparent to visible light, thus having the potential to replace potentially hazardous organic compounds in UV absorbing clear coatings. As a proof of concept, the prepared dispersions of cerium oxide nanoparticles in hexane were formulated into a solvent borne binder base to develop clear UV protecting coatings for light sensitive substrates. The general synthesis strategy presented in this study is generally applicable for the low-cost production of a concentrated dispersion of metal oxide nanoparticles with minimal environmental impact.

## Abstract 2

Solventless synthesis of metal oxide nanoparticles.

## Graphical abstract

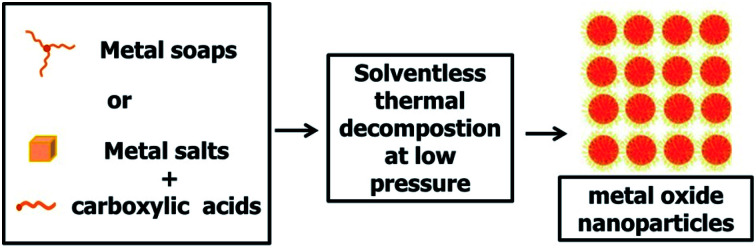

## 1. Introduction

Cerium oxide (ceria, CeO_2_, CeO_2−*x*_) nanoparticles are widely studied for their applications in catalysis,^1^ chemical and mechanical polishing (CMP), fuel cells,^2^ sun-screens,^3^ infrared reflective pigments,^4^ UV^5–7^ and corrosion protective^8–10^ coatings and bio-medicines.^11–14^ The high demand for cerium oxide nanoparticles from the CMP application especially in North America is driving the growth of the global cerium oxide nanoparticle market and the current development technology is not able to cover the increasing demand.^15^

Synthesis of colloidal metal oxide nanoparticles with controlled shape and size is of fundamental and technological interest due to their importance in understanding nanoscale physical phenomena and their various applications, such as in optics, catalysis, energy, and microelectronics.

Presently, highly dispersible metal oxide nanoparticles are synthesized using number of wet chemical methods including controlled precipitation,^16^ micro-emulsion, sol–gel and thermal decomposition of metal–organic precursors in hot organic solvents. Growth of high-quality inorganic nanoparticles *via* the latter method has become a common practice. High degree control of nanoparticles composition, size, shape, crystallinity and surface functionality is possible. However, these methods typically use a large amount of toxic and high boiling point organic solvents as a reaction medium, which makes the process expensive and difficult to separate or recover the nanoparticles after synthesis.^17–24^

Industrial scale production of dispersible nanoparticles using these processes has been costly and technically challenging because of the lack of effective methods to separate the well-dispersed nanoparticles from the reaction solvents and the difficulty of handling and recycling the liquid waste. Not surprisingly, such a liquid based colloidal nanoparticles production processes have not been yet adopted by industry for the synthesis of dispersible metal and metal oxide nanoparticles.

Solventless thermolysis of metal–organic complexes is an alternative method to overcome many of the typical problems encountered in colloidal synthesis of inorganic nanoparticles.^25–28^ An important advantage of this method is the absence of high boiling point organic solvents which substantially reduces nanoparticle production costs.

**Fig. 1:**
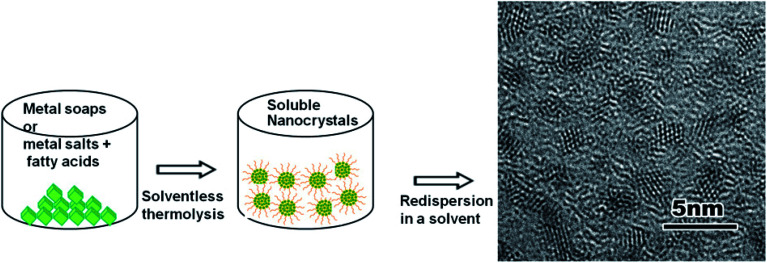
A general scheme for the solventless synthesis of solvent dispersible metal oxides.

A general synthetic route to obtain dispersible metal oxide nanoparticles by solventless thermolysis of metal-carboxylates is shown in Fig. 1. Metal-carboxylates (metal soaps) are used as molecule precursors and a thermolysis reaction is carried out in a low pressure closed vessel to produce solvent dispersible metal oxide nanoparticles.

This method generally relies on two important parameters:

(*i*) *Selection or preparation of suitable metal carboxylate precursors that can be easily decomposed at relatively low temperature*. In the case of using a physical mixture of metal salts and fatty acids, removal of the resulting insoluble salts is necessary. Most of the metal soaps or combination of metal salts with fatty acids used in traditional colloidal thermal decomposition processes can also be conveniently adapted to this process.^17,29^

(*ii*) *Low pressure thermolysis of metal-carboxylates in a closed reaction vessel*. The low-pressure in the closed reaction vessel can prevent high pressure build up and thus explosions. Closed reaction vessels can contain the organic molecules in the reaction system preventing direct release of decomposition side products into air. In industrial scale production of nanoparticles, these side products could be easily collected and treated before their release to the environment. Furthermore, by controlling the initial pressure and time of reaction system, both morphology and crystal structure of nanoparticles can be also controlled to a certain degree.^30^

Moreover, in the solventless reaction environment, nanoparticle collisions are limited and particle growth proceeds primarily by monomer addition to the particle surface, leading to monodisperse nanoparticle formation.^28,30,31^

The solventless thermal decomposition process typically generates a viscous paste of dispersible nanoparticles that can be readily mixed with low boiling point organic solvents without the need for separation, cleaning or a size-selection process. This is highly desirable for large scale production of solvent dispersible nanoparticles which can be easily re-dispersed in the desired solvent afterwards. Therefore, synthesis and stabilization cost of functional metal and metal oxide nanoparticles in a desired solvent can be greatly reduced.^32^

Nanoparticle synthesis by solventless thermolysis of metal–organic complexes has been widely used during the last two decades. Some examples worth mentioning are the monodisperse iron oxide nanoparticles synthesis by thermal decomposition of iron-oleate at low pressure conducted by Cha *et al.*^30,31^ and the formation of Cu nanoparticles by thermal decomposition of Cu-oleate powders at low pressure developed by Kim *et al.*^31^ Moreover, Han *et al.* reported the synthesis of Fe nanoparticles by the thermal decomposition of iron-oleate in the presence of NaCl,^33^ Kim *et al.* synthesized copper nanoparticles by thermal decomposition of copper-oleate in high pressure autoclaves^31^ and Pan *et al.* performed the synthesis of dispersible nanoparticles of MgO, Ga_2_O_3_, In_2_O_3_ and GaIn_2_O_3_ in open reaction vessels.^34^

Therefore, it has been a considerable effort to generate different types of nanoparticles by solventless thermolysis. However, cerium oxide nanoparticles, despite their undeniable relevance, have not been the target of a systematic study yet.

Thus, a more efficient and cost-effective synthesis method for dispersible cerium oxide nanoparticles must be developed. Here, a new simple solvent-free thermolysis route is proposed to synthesize dispersible cerium oxide nanoparticles that can be potentially used at an industrial scale.

## 2. Experimental

### 2.1. Materials

Sodium oleate (97%) was purchased from Riedel-de Haën (Seelze, Germany). Anhydrous *n*-hexane (95%) and Ce(NO_3_)_3_·6H_2_O (99.9%) were supplied by Sigma Aldrich (Taufkirchen, Germany). Deoxygenated water was prepared by bubbling N_2_ through double distilled deionized water. Pyrex tubes (7 mm light wall with a 750 mm length) were purchased from Saveen Werner (Mälmo, Sweden). An Edwards RV8 vacuum pump and Active Pirani Gauge Controller were obtained from Edwards Vacuum (Albany, NY, USA). Freeze drying equipment was obtained from Amsco Finn-Aqua (Hurth, Germany).

### 2.2. Synthesis of cerium-oleate

To prepare the cerium oleate precursor, sodium oleate and cerium nitrate salt solutions were first prepared. The sodium oleate solution was obtained by dissolving 9.13 g of sodium-oleate (30 mmol) in 300 ml of deoxygenated water. The cerium nitrate solution was prepared using 3.44 g of Ce(NO_3_)_3_·6H_2_O (10 mmol) dissolved in 100 ml of deoxygenated water. Immediately after preparation, these solutions were covered with parafilm and stirred for 30 min. Afterwards the salt solution was added dropwise to the sodium-oleate solution and the resulting suspension was sealed and stirred with a magnetic stirrer for 2 hours to obtain a white suspension. This suspension was then centrifuged at 3000 rpm for 20 minutes to a white precipitate (see Fig. SI-1†). The precipitate was then washed several times with deoxygenated water until the pH was below 8 and subsequently filtered. The collected wet cake of cerium-oleate was freeze-dried for three days to obtain free-flowing, fluffy white powders of cerium-oleate. Samples prepared in this way were sealed in a nitrogen atmosphere and stored in a refrigerated, oxygen-free atmosphere until used in further experiments.

### 2.3. Synthesis of dispersible cerium oxide nanoparticles

13 mg of the previously synthesized cerium oleate powders were placed in a 12 cm long, tubular Pyrex ampoule. The ampoule was then evacuated at 0.3 mbar using a vacuum pump and carefully sealed with a butane torch. The resulting ampoule was wrapped in aluminum foil and placed horizontally in an oven. The sample was then heated at 320 °C for different periods of time in a pre-heated oven (see Fig. SI-2†). After the reaction, the ampoule was cut and the resultant thermally decomposed product was dissolved in 2 ml of hexane and sonicated gently for 5 minutes obtaining a hexane dispersion of colloidal ceria nanoparticles.

### 2.4. Characterization

X-ray photoelectron spectroscopy (XPS) was performed using a Kratos AXIS UltraDLD XPS (Kratos Analytical, Manchester, UK). The samples were analyzed using a monochromatic Al X-ray source. Thermal gravimetric data were collected using a Pyris 1 TGA (PerkinElmer, Waltham, Massachusetts, USA). The samples were placed in a platinum crucible under N_2_ atmosphere with a heating rate of 5 °C min^−1^ and a maximum temperature of 750 °C. Real time thermal decomposition of cerium-oleate was studied with the Pyris 1 TGA coupled to a FT-IR (Spectrum one, PerkinElmer, Waltham, MA, USA) to obtain real time FT-IR spectra of the decomposition product of cerium-oleate under nitrogen atmosphere with a heating rate of 5 °C min^−1^ and a maximum temperature of 350 °C. Differential scanning calorimetric (DSC) data were collected using a DSC 1 STAR System Thermal Analysis (Mettler Toledo, Gießen, Germany). The powders were heated in platinum cups under N_2_ atmosphere with a heating rate of 5 °C min^−1^ and a maximum temperature of 600 °C. A FT-IR-Spectrometer, “Spectrum One” with Universal Attenuated Total Reflection (ATR) sampling accessory (PerkinElmer, Waltham, MA, USA) was used with a MIR (mid-infrared) beam source. The ATR crystal was made of diamond and the detector utilized was a MIR-DTGS (mid-infrared deuterated triglycine sulfate). For liquid samples, one drop of solution was placed on the ATR crystal and for powder specimens, a few milligrams of powders were placed directly on the ATR crystal. Powder XRD (PXRD) patterns were obtained using a PANalytical X'Pert PRO Alpha-1 system (Malvern Panalytical, Malvern, United Kingdom) working with Cu Kα1 radiation with wavelength 1.54 Å. UV-VIS spectra of the samples were collected with a Elmer Lambda-650 spectrometer (PerkinElmer, Waltham, MA, USA). The two light sources used were a deuterium lamp (UV-VIS range) and a halogen lamp (near infrared range). Hexane-resistant polyamide or quartz curettes were used as sample holders. The ceria nanoparticle hexane dispersions were diluted by a factor of 2000 in hexane for the analyses. Transmission electron microscopy (TEM) was performed using a JEM-3010 (JEOL, Akishima, Japan). TEM samples were prepared by evaporating hexane from one drop of ceria nanoparticle dispersion placed on a carbon coated copper grid. Solvent borne clear coating were obtaining by adding 10 wt% of a ceria nanoparticles hexane dispersion (see Fig. SI-3†) into the alkyd resin Setal 291 (Akzo Nobel, Amsterdam, Netherlands) obtaining different ceria nanoparticle concentrations of 2.0 wt%, 1.0 wt% and 0.0 wt%. These coating formulations were applied on pre-cleaned quartz slides using a 100 μm thick film applicator resulting in roughly 50 μm thick transparent dry films. The UV absorption spectrum of the 2 wt% film was carried out using a JASCO V-650 spectrophotometer (Jasco, Pfungstadt, Germany) with a 150 mm diameter integrating sphere.

## 3. Results and discussions

The thermal decomposition of cerium oleate is an important step in synthesizing dispersible ceria nanoparticles. There are many parameters involved that need to be considered. The initial part of this investigation is to narrow down the range of temperature needed to synthesize dispersible cerium oxide nanoparticles with uniform size. In order to investigate this, a systematic study using TGA, DSC, FTIR, XRD and TEM have been performed.

**Fig. 2:**
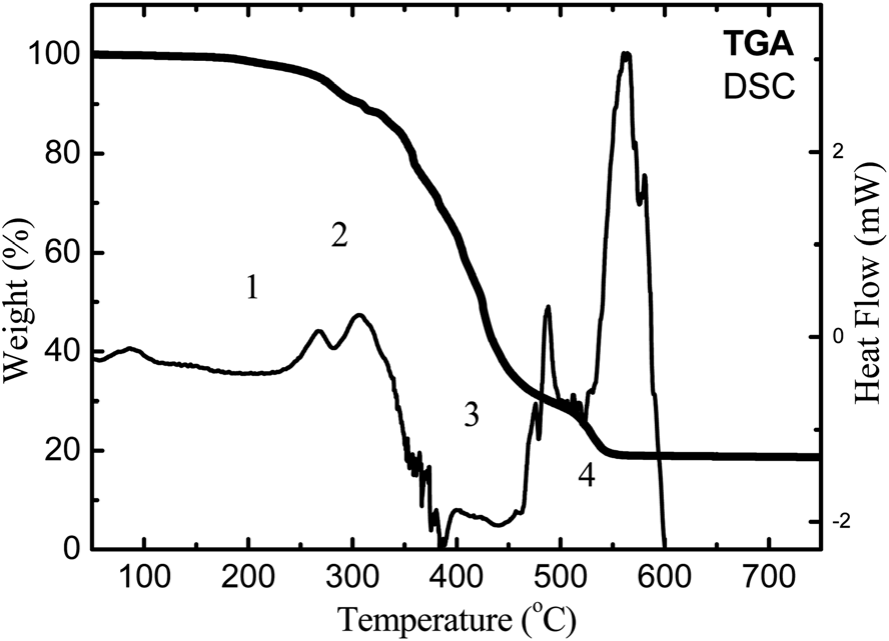
DSC-TGA plot of cerium oleate thermal decomposition.

Fig. 2 shows TGA and DSC curves for pure cerium-oleate showing four distinct steps during thermal decomposition of cerium oleate. The first step represents the evaporation of the water absorbed on the freeze-dried cerium-oleate precursor. This event occurred between 20 and 200 °C and led to a weight loss of about 2 wt%.

The second step, with a weight loss of about 9 wt%, corresponds to the decomposition of the cerium-oleate precursor chains between 200 and 300 °C. The two exothermic peaks observed in the DSC curve between 250 and 300 °C were generated during the decomposition of the acyl groups without formation of the metal oxide.^35^

**Fig. 3:**
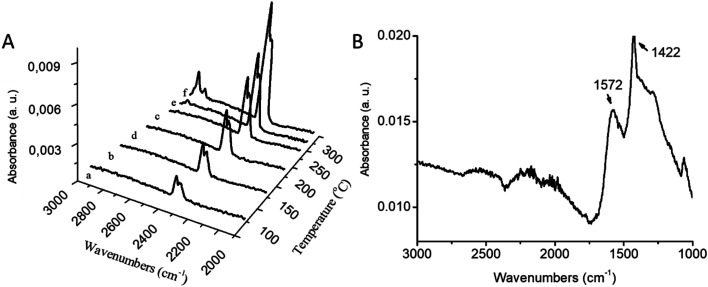
FTIR-TG plots of cerium oleate decomposition at various temperatures (A) (a) 120 °C; (b) 170 °C; (c) 220 °C; (d) 270 °C; (e) 295 °C; (f) 320 °C and (B) 520 °C, both measurements were performed in nitrogen atmosphere.

Coupled FTIR-TGA spectra of cerium oleate and their decomposition products shown in Fig. 3A displays the carbon dioxide (2360–670 cm^−1^) and hydrocarbon gases (anti-symmetric and symmetric stretches of CH_2_ at 2937 and 2860 cm^−1^) signals around 295 °C (Fig. 3A). The detection of all these products is consistent with the decomposition of the oleate chain at these high temperatures.

During the third step between 320 and 465 °C a significant endothermic reaction is observed in the DSC curve (Fig. 2), indicating the formation of the metal oxide ceria.^35^ Also at this temperature range, two major loses are clearly visible in the TGA curve (Fig. 2) which sum up to a considerably summed weight-loss of 56 wt%. Kenfack and Langbein^35^ related these two peaks to the release of carbon monoxide (first) and carbon dioxide (second) due to the partial decomposition of the oleate chain. According to their explanation, when the temperature is slowly increased, carbon monoxide is first released, and further heating leads to the formation of carbon dioxide. However, in Fig. 3A, the FTIR-TGA investigation was only able to detect carbon dioxide in the gaseous decomposition products with no indication of the presence of carbon monoxide.

The fourth and final step occurred over 456 °C. In this temperature range a weight loss of 13.5 wt% and an exothermic process (assigned to an oxidation step of Ce^3+^ to Ce^4+^) were detected in the TGA and DSC curves, respectively (Fig. 2). Fig. 3B shows the FTIR spectrum of the cerium oleate sample heated at 520 °C displaying the two characteristic peaks of metal carboxylate at 1572 and 1442 cm^−1^.^28,36^ While these peaks may suggest that the oleate chain has not been consumed, the absence of the vibration peaks of CH_2_ around 2900 cm^−1^ indicates that the acyl groups were decomposed, and thus responsible for the weight loss and exothermic peaks observed in TGA and DSC, respectively.

After 550 °C, no weight loss was observed, indicating that the decomposition and volatilization of the organic fraction was already completed. However, the exothermic events continue until 600° when all the Ce^3+^ was slowly converted into Ce^4+^.

The temperature decomposition path presented here is characteristic of cerium oleate. For other organic coordinated metal compounds, the path is controlled by the structure of the organic chain and the coordinated metal. Some metal–oxygen bonds can be decomposed at temperatures lower than 300 °C and others remain stable up to 520 °C.^28,37,38^

**Fig. 4:**
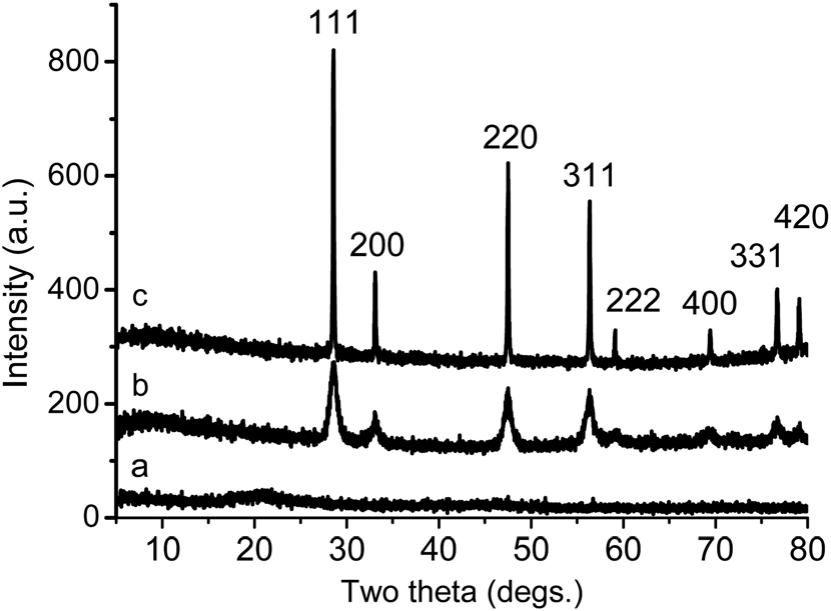
XRD patterns of the decomposition products of cerium oleate at different temperatures: (a) 20 °C; (b) 520 °C; (c) 750 °C.

The powder X-ray diffractograms in Fig. 4 show three distinct phases in the growth of ceria during this process. At 20 °C, the XRD pattern of cerium-oleate displays only a single, broad peak corresponding to the disordered packing of the oleate chains and no crystalline ceria is observed. After heating to 520 °C and subsequent cooling, the chain-packing peak disappears, while relatively broad peaks appear indicating the growth of face-centered cubic (*Fm*3̄*m*) ceria. After cooling from 750 °C sharp diffraction peaks due to scattering from relatively large crystallites of face-centered cubic ceria are clearly visible.

The temperature to synthesize dispersible nanoparticles with uniform size depends on the thermal degradation of the metal-carboxylate precursor.^28^ Thus, the temperature should be high enough to provide an effective concentration of intermediates moieties, but low enough to prevent the complete degradation of the oleate chains^39^ and the incontrollable growth of the nanoparticles. Moreover, the selection of the stabilizer is also an extremely important factor in obtaining monodisperse ceria nanoparticles. The oleate chain in the synthesis of dispersible ceria nanoparticles is a crucial factor because of the *cis*-double bond in the middle of the chain. This bond forms a kink which is considered necessary for the effective dispersion of the nanoparticles. For example, stearic acid, which does not have this kink, is not able to successfully stabilize suspensions.^40^

Therefore, based on the data acquired above, the thermal decomposition of cerium oleate and growth of ceria was performed at 320 °C.

A series of experiments were designed to thermally decompose cerium oleate at an arbitrary low pressure of 0.30 mbar at 320 °C.

**Fig. 5:**
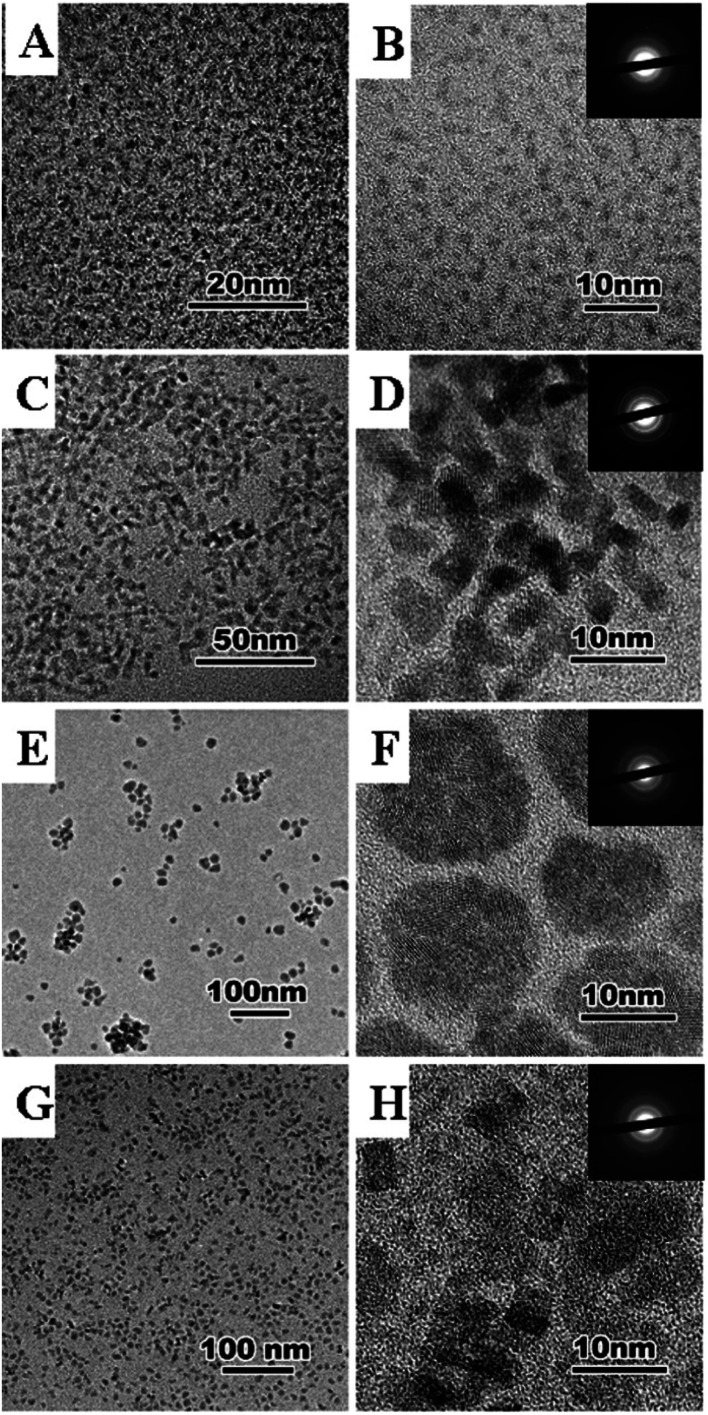
TEM images and selected area diffraction patterns of ceria nanoparticles synthesized by thermolysis of cerium oleate at 0.3 mbar and 320 °C for different times. 0.7 h, 0.3 mbar (A and B); 24 h, 0.3 mbar (C and D); 72 h, 0.3 mbar (E and F); 144 h, 0.3 mbar (G and H).

Fig. 5 shows the evolution of the oleate coated cerium oxide nanoparticles as a function of time at 320 °C. Decomposition products were released with time and ceria nanoparticles with different shapes and sizes were obtained (Fig. 5 and Table 1). Monodisperse ceria nanoparticles with an average size of 2–3 nm were observed after 0.7 hours (Fig. 5A and B). When, the decomposition time was extended to 24 hours, single-crystal ceria nanoparticles with rectangular shape were obtained with diameters of 8–10 nm (Fig. 5C and D). An increase of the decomposition time to 72 hours led to the formation of polycrystalline spherical nanoparticles with average size of 15–20 nm (Fig. 5E and F). Keeping the sample at 320 °C for 144 hours led to the formation of single crystalline faceted nanoparticles with average size of 4–8 nm (Fig. 5G and H). Therefore, it can be concluded that the ceria nanoparticles growth is governed by a nucleation–dissolution–recrystallization growth mechanism,^40^ which leads to ceria nanoparticles with different shape and size depending on the reaction time.

**Table 1:** Effect of different reaction times in cerium oleate nanoparticles size and shape

Time (hours)	Ceria nanoparticles	Size (nm)
0.7	Spherical/rectangular	2–3
24	Rectangular	8–10
72	Polycrystalline spherical	15–20
144	Rectangular	4–8

**Fig. 6:**
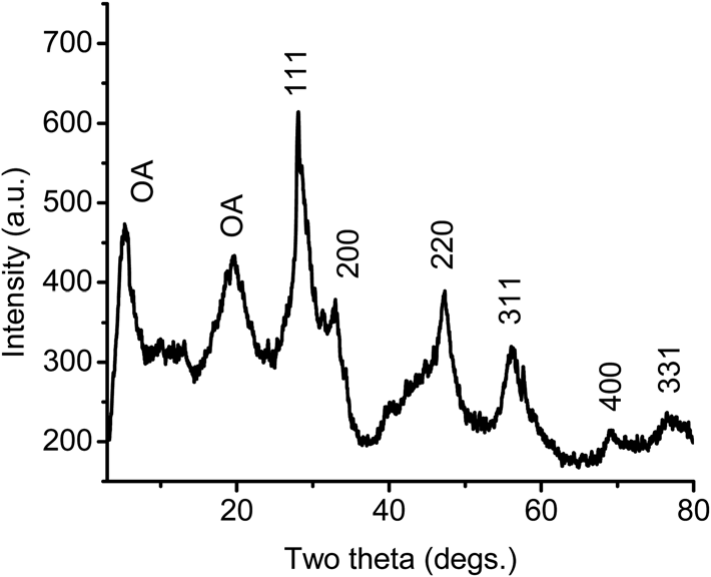
XRD pattern of ceria nanoparticles synthesized at 320 °C, 0.7 h, and 0.3 mbar.

**Fig. 7:**
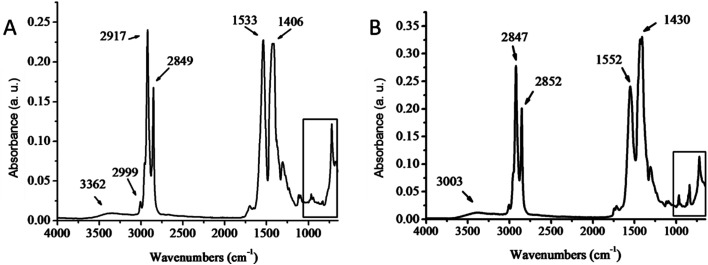
FT-IR spectra of as synthesized ceria nanoparticles 320 °C, 0.7 h and 0.3 mbar (A) and as synthesized cerium oleate (B).

Monodisperse ceria nanoparticles obtained after 0.7 hours of heating at 320 °C and 0.3 mbar (2–3 nm, Fig. 5A and B) were separated from each other by approximately 4 nm due to the characteristic stearic repulsion between the oleic acid chains attached to the ceria nanoparticles. XRD patterns of the as-prepared cerium oxide sample shows in Fig. 6 characteristic peaks of both oleate chains (OA) and the cubic (fluorite) structure of ceria that indicate the formation of nano-ceria capped with oleate ligands. Broad diffraction peaks in Fig. 6 and diffused diffraction rings in the inset of Fig. 5B reveal the nanocrystalline nature of the sample obtained at 320 °C. The crystal size calculated from the XRD pattern using the Scherrer equation^41^ is 4.38 nm and agreed well with the TEM observation (Fig. 5A and B). In addition, the first peak located at around 5.328° in XRD pattern (Fig. 6) presents a *d*-spacing of 16.54 Å, which is probably related to the self-organization and assembly of the alkyl chain between ceria nanoparticles.^42^ The existence of oleate ligand bound to synthesized ceria nanoparticles is supported by the FT-IR spectra shown in Fig. 7. Fig. 7A displays the FTIR spectrum of the obtained oleate coated ceria where the two signals observed at 1552 and 1430 cm^−1^ corresponds to the carboxylate group attach to the ceria. These signals are generated by the ionic interaction between the anionic carboxylate group and the cationic metal surface.^28,36^ Moreover, the carboxylate absorption bands produced by the COO^−^ deformation, bending and wagging are observed below 1000 cm^−1^. All of these suggest the carboxylate groups coating the ceria remain stable at 320 °C.^28,36^

Fig. 7A shows also at 2847 and 2852 cm^−1^ the symmetric and asymmetric stretches of –CH_2_.^36^ These signals suggest that the aliphatic part of the oleate chain does not decompose either when the ceria nanoparticles are formed.

As it was described above the unsaturated double bound located in the oleate chain is crucial to ensure the creation of monodisperse nanoparticle suspensions, thus the verification of the stability of this bond at the reaction temperature is important in the synthesis of oleate-coated ceria nanoparticles. In Fig. 7A the small vibration band near 3000 cm^−1^ corresponds to the unsaturated C_9_–C_10_ double bond^36^ and suggests that at 320 °C this bond is still stable.

Fig. 7B shows the FT-IR spectrum of the cerium oleate used as the starting material in the synthesis of the coated ceria nanoparticles. The peaks displayed in this figure almost match the peaks of the coated ceria in Fig. 7A. The unique differences observed between both spectra are shifts in the main oleate peaks presented before. These shifts are due to the rearrangements of the oleate chains after the transition from cerium oleate to the oleate coated ceria nanoparticles. Despite the changes observed between both FT-IR spectra, the fact that the oleate main peaks remain after the reaction reveals that a fraction of the oleate chains remain relatively undisturbed even after the decomposition of cerium oleate at 320 °C.

Finally, the broad peak around 3000–3500 cm^−1^ shows the presence of the O–H stretch, due to the presence of compounds such as absorbed water, alcohol or acid. This signal, which is relatively low in Fig. 7A and B indicate the hydrophobic nature of the coated nanoparticles and the effectiveness of the freeze-drying method to eliminate the water from the cerium oleate powder.

The efficiency of this solventless process was calculated based on one of the ampoules giving a yield of about 70% (see ESI† for more details). This makes the solventless process one of the methods with the highest yield to synthesize dispersible ceria nanoparticles.

**Fig. 8:**
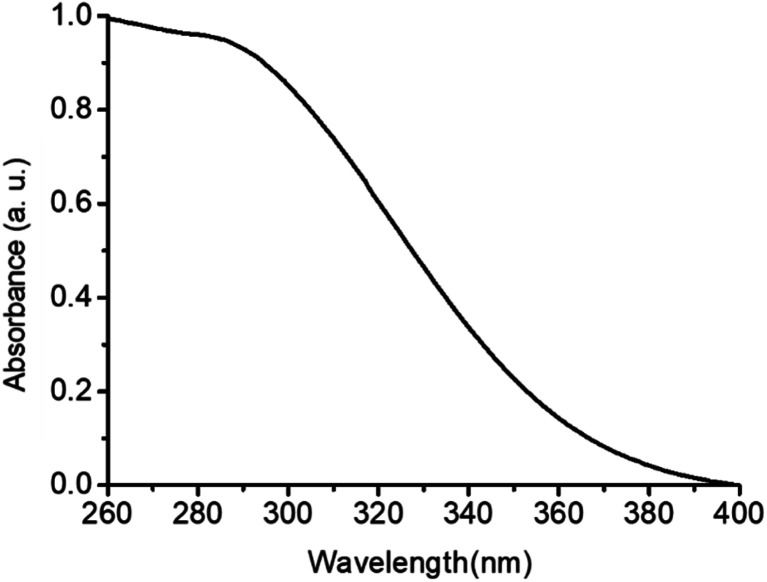
UV-VIS absorption spectrum of the ceria nanoparticles hexane dispersion synthesized at 320 °C, 0.7 h and 0.3 mbar.

Fig. 8 displays the UV-VIS absorption properties of the synthesized oleate coated ceria nanoparticles. A broad peak is observed between 250 and 370 nm which corresponds to the ultraviolet range. This indicates that the ceria nanoparticles obtained are potential UV absorbers.

**Fig. 9:**
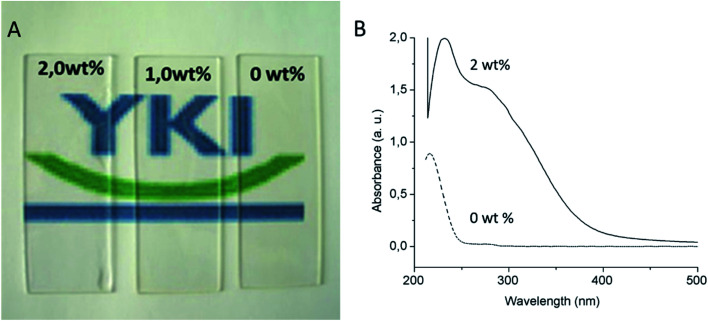
Appearance of 50 μm thick films with different ceria nanoparticle content (A) (ceria contents of the dried film are embedded into the image). UV-VIS absorption spectra of the 0 and 2 wt% dried films (B).

In order to investigate the UV protection ability of the cerium oxide nanoparticles, UV protective coatings with cerium oxide nanoparticles with different cerium oxide nanoparticle content (1.0 and 2 wt%) were formulated by adding calculated amounts of a 10 wt% hexane dispersion of cerium oxide nanoparticles (Fig. SI-3†) into a commercial solvent borne alkyd resin (Setal 291 from Akzo Nobel). The resulting mixtures were applied on glass slides forming optically clear 50 um thick dried films (Fig. 9A). The 2 wt% thick film was subsequently evaluated to verify if it maintained the UV absorption properties of the synthesized nanoparticles. Fig. 9B shows that the thick film displayed the same broad absorption peak in the ultraviolet range than the nanoparticles in Fig. 9A. This suggests that the clear coating developed in this work with ceria nanoparticles could potentially replace the currently available UV absorbing clear coatings containing hazardous organic UV fillers.^29^ Further outdoor weathering test of such clear coating in UV protection of wood is under investigation and will be the topic of following paper.

## 4. Conclusion

A solventless thermolysis route for producing highly dispersible colloidal nanoparticles of cerium oxide has been proposed and experimentally verified. It was found that the thermolysis of cerium-oleate at low pressure is a suitable method for the production of highly dispersible cerium oxide nanoparticles at a relatively low cost without using any toxic high boiling point organic solvent. By tuning the temperature and time, 4 nm monodisperse, and solvent dispersible ceria nanoparticles can be easily obtained.

Dispersions of the synthesized nanoparticles in hexane showed excellent absorption properties in the UV wavelength range and thus can be used as UV absorbing additives for UV protective clear coatings. The development of clear coatings with a permanent UV absorber, such as ceria could be used to protect materials from long term UV degradation, such as wood and could potentially replace the currently available organic UV absorbers in clear coatings which typically degrade easily under UV irradiation within 2 years.

The general synthesis strategy presented in this study is generally applicable for the low-cost production of concentration dispersion of metal oxide nanoparticles with a minimum environmental impact.

## Conflicts of interest

There is no conflict of interest.

## Supplementary Material

RA-010-D0RA01710H-s001
