# Supplementary material for: Solventless synthesis of cerium oxide nanoparticles and their application in UV protective clear coatings
Source: RSC Adv. 2020 Apr 14;10(25):14818–25. doi: 10.1039/d0ra01710h (PMC9052101; doi:10.1039/d0ra01710h)
Supplement: RA-010-D0RA01710H-s001 [file RA-010-D0RA01710H-s001.pdf]

Electronic Supplementary Material (ESI) for RSC Advances.
This journal is © The Royal Society of Chemistry 2020

Supplementary information

Solventless synthesis of cerium oxide nanoparticles and its application in UV protective clear coating

Rubén Álvarez Asencio[a], Robert W. Corkery[b], Anwar Ahniyaz[a]*,
a RISE Research Institutes of Sweden, Division of Bioscience and Materials, SE-114 86 Stockholm, Sweden.
b KTH Royal Institute of Technology, School of Engineering Sciences in Chemistry, Biotechnology and Health.

* Corresponding author. E-mail: anwar.ahniyaz@ri.se

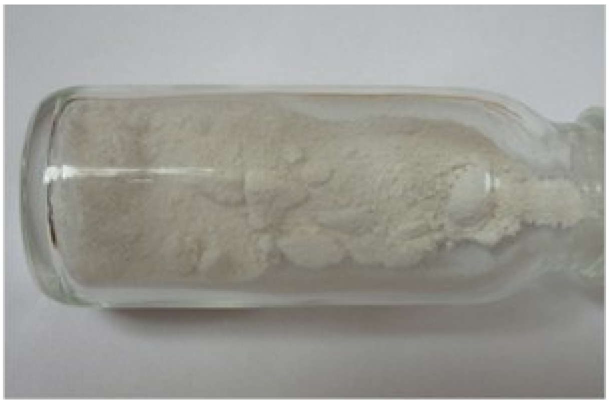

SI-1. Photograph of white cerium oleate powder.

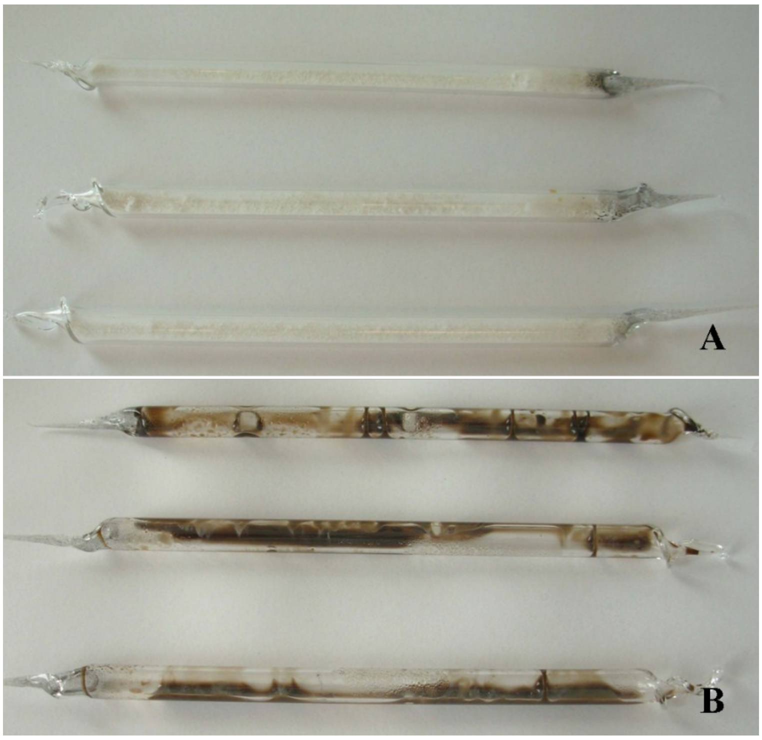

SI-2. Snapshots of three samples before (A) and after (B) thermal decomposition of cerium oleate at 320 ˚C for 0.7 h at 0.3 mbar.

The yield of this method was calculated based on one of the ampoules. The ampoule containing 0.13 g of cerium oleate (0.000132 mol, 945.5 gr/mol) yielded 0.05 g of oleate coated ceria after the thermal decomposition described above. These 0.05 g were burned at 600 ºC for 1 hour to remove the organics resulting into 0.016 g of cerium dioxide (172.12 g/mol). Theoretically, as the relation between ceria and cerium-oleate is 1:1 (molar), 0.13 g of cerium-oleate should generate 0.023 g of CeO_2. Equations (1) and (2) show the calculations needed to calculate the yield. M is the molecular mass (g/mol), $m_{ceria,t}$ is the ceria theoretical mass (g), $m_{sample,0}$ is the initial sample mass of the cerium-oleate (g) and $m_{ceria,e}$ is the ceria experimental mass (g) obtained after thermal decomposition (g). Thus, considering that no side products were obtained, the yield of this solventless process was about 70 %.

$$m_{ceria,t} = \frac{M_{ceria}}{M_{cerium\text{ - }oleate}} m_{sample,0}$$

(1)

$$Y = \frac{m_{ceria,t}}{m_{ceria,e}} \cdot 100\%$$

(2)

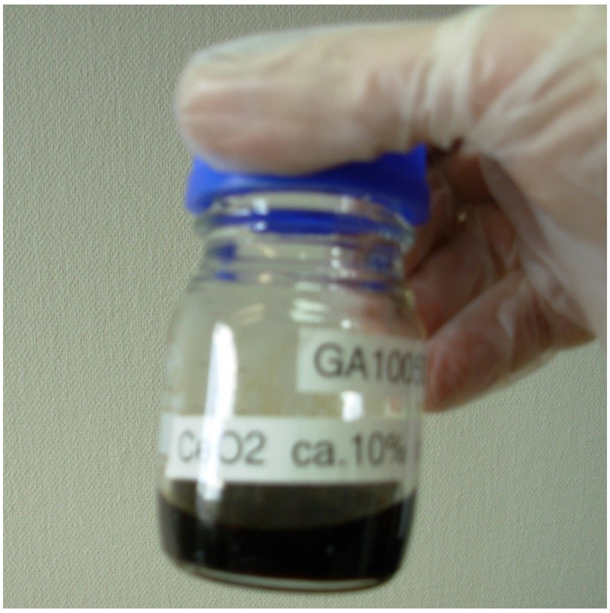

SI-3. Photograph of 10 wt% hexane dispersion of cerium oxide nanoparticles prepared at 320°C, 0.7 h and 0.3 mbar.

SI-3 shows a 10 wt% hexane dispersion of ceria nanoparticles with a dark brownish color. No precipitation and no phase separation were observed after storing the dispersion for more than 3 years in the lab.
